# Association between previous spontaneous abortion and preeclampsia: a case–control study

**DOI:** 10.1186/s12884-022-05053-8

**Published:** 2022-09-19

**Authors:** Ahmed Mohamedain, Duria A. Rayis, Nadiah AlHabardi, Ishag Adam

**Affiliations:** 1 Department of Obstetrics and Gynaecology, Faculty of Medicine, University of Khartoum, Khartoum, Sudan; 2 Department of Obstetrics and Gynecology, Unaizah College of Medicine and Medical Sciences, Qassim University, Unaizah, Kingdom of Saudi Arabia

**Keywords:** Spontaneous abortion, Preeclampsia, Risk factors, Pregnancy

## Abstract

**Background:**

The association between previous spontaneous abortion and preeclampsia is not yet fully understood. The current study was conducted to assess the association between previous spontaneous abortion and preeclampsia among pregnant women in Sudan.

**Methods:**

A case–control study (involving 180 women in each study group) was conducted at Saad Abuelela Hospital, Khartoum, Sudan. The cases were pregnant women with preeclampsia, while the control group included healthy pregnant women. The participants’ sociodemographic, obstetric, and clinical characteristics were assessed via a questionnaire.

**Results:**

There was no significant difference in the age, parity, education level, employment status, blood group, body mass index, and hemoglobin level between the patient and control groups. Forty (22.2%) women with preeclampsia and 68 (37.8%) women in the control group had a history of spontaneous abortion (*p* = 0.001).

Multivariate logistic regression analysis (adjusted) revealed that women with a history of spontaneous abortion had a lower risk of preeclampsia than those without a history of spontaneous abortion [adjusted odds ratio (AOR) = 0.44, 95% confidence interval (CI) = 0.26‒0.73]. However, women with a history of preeclampsia had a higher risk of recurrence of preeclampsia (AOR = 1.92, 95% CI = 1.11‒3.32).

**Conclusion:**

The present study revealed that previous spontaneous abortion reduced the risk of preeclampsia by 59.0%.

## Introduction

Preeclampsia is a multi-pathway disorder that is characterized by new-onset hypertension and proteinuria in the second half of pregnancy (i.e., after the 20th week of gestation); it is one of the most common pregnancy-related disorders [1]. Preeclampsia is a global health challenge, affecting 2%–8% of pregnant or parturient women. It can have maternal and perinatal adverse effects and is the leading cause of maternal and perinatal morbidity and mortality [2]. Despite advances in various sciences to understand the pathophysiology of preeclampsia, its exact cause and etiology remain unclear. Previous research has revealed several sociodemographic, obstetric, clinical, biochemical, and genetic factors associated with preeclampsia [3]. We previously found that preeclampsia/eclampsia was responsible for 4.2% of the admissions (total admissions = 4689) to a maternity hospital in Kassala in eastern Sudan and that preeclampsia was the main cause of maternal and perinatal morbidity and mortality [4].

Several studies have reported an association between prior abortion and preeclampsia [5–16]. However, the results of these studies are inconsistent; while some studies have reported an increased risk of preeclampsia in women with a history of abortions [5–8], others have reported no such effect [9–12] or a reduced risk of preeclampsia in women with a history of abortions [13–16]. Moreover, most these studies have been conducted in high-income countries [7, 9, 13, 16]. There is a scarcity of data on the history of abortion and preeclampsia in sub-Saharan African countries [6], and none of these studies have been conducted in Sudan. The risk factors for preeclampsia may differ in different populations. It is therefore necessary to assess these risk factors in different populations. It is important for clinicians, researchers, and health planners to identify the risk factors (including history of spontaneous abortion) for preeclampsia in various settings in order to identify women who are at a higher risk of preeclampsia and to provide optimum care.

The present study aimed to assess the association between previous spontaneous abortion and preeclampsia among pregnant women in Sudan.

## Methods

### Study design and setting

The present case–control study was conducted at Saad Abuelela Hospital, Khartoum, Sudan, from February to December 2020.

### Selection of the participants

The patient group included consecutive pregnant women who presented with preeclampsia during the aforementioned period. Preeclampsia was defined as follows, according to the criteria of the American College of Obstetricians and Gynecologists [1]: (1) average blood pressure levels ≥ 140/90 mmHg on two readings taken at least 6 h apart and (2) proteinuria ≥ 300 mg/24 h in pregnant women. Preeclampsia was considered to be severe in women with average blood pressure levels ≥ 160/110 mmHg on two occasions or proteinuria ≥ 5 g/24 h, in addition to hemolysis, elevated liver enzymes, and low platelet count (HAELLP) syndrome; in all other cases, preeclampsia was considered to be mild [1]. Moreover, preeclampsia that developed before and after 34 weeks of gestation was defined as early-onset and late-onset preeclampsia, respectively [17]. A healthy pregnant woman (consecutively to each woman with preeclampsia) without any systemic disease, such as hypertension, thyroid disease, severe anemia (hemoglobin level < 5 g/dl), diabetes mellites, renal disease, or proteinuria, served as a control for each patient with preeclampsia. Women with multiple pregnancies, who were smokers, or whose fetuses had major congenital anomalies were excluded from both the cases and controls.

After the participants signed an informed consent form, they were asked about their sociodemographic, obstetric, and clinical characteristics (such as age, parity, education level, employment status, blood group, history of abortion, history of preeclampsia, and gender of the newborn). The participants’ weight and height were measured using standard procedures to compute the body mass index (BMI) [18]. The hemoglobin level was measured using an automated hematology analyzer, according to the manufacturer^’^s instructions (Sysmex, KX-21, Japan).

### Sample size

A total of 180 women were included in each study group (a ratio of 1:1); the sample size was calculated on the basis of previous data on the history (43.0%) of spontaneous abortions among women with preeclampsia in neighboring Ethiopia [6]. Therefore, we assumed that 40.0% of women with a history of spontaneous abortion would have preeclampsia and 25.0% of women with a history of spontaneous abortion would not have preeclampsia. This sample size (180 in each study group) was used to achieve 80% power and 5% precision; it was assumed that 10% of the women would not respond or would have incomplete data.

### Statistical analysis

Statistical analysis was performed using Statistical Package for the Social Sciences® (SPSS®; IBM SPSS Statistics for Windows, version 22.0; SPSS Inc., New York, United States). The proportions of the studied variables are expressed as frequencies (%). The normality of continuous data was assessed using the Shapiro–Wilk test. Multicollinearity (variance inflation factor < 4) was assessed but was not detected among the variables. Associations between specific variables, such as age, parity, education level, employment status, blood group, history of preeclampsia, history of miscarriage, BMI, gender of the newborn, and preeclampsia, were assessed using univariate analysis. Variables with a *p*-value ≤ 0.200 in univariate analysis were selected for the construction of multivariate models that considered crude associations between preeclampsia and the variables. Backward elimination (likelihood ratio) was used to adjust the model for covariates. Adjusted odds ratios (AORs) and 95% confidence intervals (CIs) were calculated. A two-sided *p*-value ≤ 0.050 was considered to denote statistical significance.

## Results

During the study period, 117 (65.0%) and 63 (35.0%) women presented with mild and severe preeclampsia, respectively. There was no significant difference in the age, parity, education level, employment status, blood group, BMI, hemoglobin level, and gender of the newborn between the patient and control groups (*n* = 180 in each). The number of women with a history of spontaneous abortion was lesser in the patient group than in the control group. Forty (22.2%) women with preeclampsia and 68 (37.8%) women in the control group had a history of spontaneous abortion (*p* = 0.001). A higher number of women with preeclampsia had a history of preeclampsia (Table 1).

**Table 1:** Univariate analysis of sociodemographic and clinical variables among women with preeclampsia and controls at Saad Abuelela Hospital, Khartoum, Sudan, in 2020

Variables	Women with preeclampsia (*n* = 180)	Controls (*n* = 180)	OR (95% CI)	*p*-value
**Age, years**^**a**^	28.5 (7.0)	29.0 (7.0)	1.01 (0.96‒1.04)	0.884
**Parity**^**a**^	3.0 (3.0)	2.0 (2.0)	1.11 (0.99‒1.24)	0.072
**Body mass index, kg/m**^**2**^	24.9 (3.2)	24.9 (3.3)	1.00 (0.91‒1.09)	0.999
**Hemoglobin level, g/dl**	10.5 (1.7)	10.5 (2.5)	0.89 (0.77‒1.02)	0.102
**Education level**				
Secondary or higher	133 (73.9)	140 (77.8)	Reference	
Less than secondary	47 (26.1)	40 (22.2)	1.25 (0.76‒2.04)	0.389
**Employment status**				
Housewife	168 (93.3)	167 (92.8)	Reference	
Employed	12 (6.7)	13 (7.2)	0.91(0.40‒2.06)	0.836
**History of preeclampsia**				
No	135 (75.0)	146 (81.1)	Reference	
Yes	45 (25.0)	34 (18.9)	1.43 (0.86‒2.36)	0.162
**History of miscarriage**				
No	140 (77.8)	112 (62.2)	Reference	
Yes	40 (22.2)	68 (37.8)	0.47 (0.29‒0.74)	0.001
**Blood group**				
Blood group type O	70 (38.9)	74 (41.1)	Reference	
Blood group type other than O	110 (61.1)	106 (58.9)	1.09 (0.71‒1. 67)	0.667
**Gender of the newborn**				
Female	104 (57.8)	97 (53.9)	Reference	0.458
Male	76 (42.2)	83 (46.1)	1.14 (0.78‒1.73)	

*CI* Confidence interval, *OR* Odds ratio

^a^ Median (interquartile range)

Multivariate logistic regression analysis (adjusted) revealed that women with a history of spontaneous abortion had a lower risk of preeclampsia than those without a history of spontaneous abortion (AOR = 0.44, 95% CI = 0.26‒0.73, *p* < 0.001). However, women with a history of preeclampsia had a higher risk of recurrence of preeclampsia (AOR = 1.92, 95% CI = 1.11‒3.32, *p* = 0.022, Table 2).

**Table 2:** Multivariate analysis of the adjusted odds ratios for the factors associated with preeclampsia at Saad Abuelela Hospital, Khartoum, Sudan, in 2020

**Variables**		**Adjusted values**		
		**OR**	**95% CI**	***p*-value**
Parity		1.04	0.92‒1.17	0.513
Hemoglobin level		0.90	0.78‒1.04	0.181
History of preeclampsia	No	Reference		
	Yes	1.92	1.11‒3.32	0.019
History of miscarriage	No	Reference		
	Yes	0.44	0.26‒0.73	0.002

## Discussion

The present study revealed that a history of spontaneous abortion reduced the risk of preeclampsia by 59.0% (AOR = 0.41). This finding is in accordance with that of Lao et al., who reported that prior abortion reduced the risk of preeclampsia by 15.0% among primiparous women in China (adjusted relative risk = 0.85) [14]. Similarly, Su et al. reported that a history of induced abortion was associated with a lower risk of preeclampsia among nulliparous women in China [15]. Moreover, as per a report in Norway, although two or more induced abortions reduced the risk of preeclampsia by 64.0%, one induced abortion marginally/moderately reduced the risk of preeclampsia (95% CI = 0.69–1.02] [16]. In the United States of America (Boston), Parker et al. observed a 10% reduction in the risk of preeclampsia (OR = 0.9) in women with a history of one induced abortion and a 30% reduction (OR = 0.7) in women with a history of three or more induced abortions [13]. Women with a history of abortion have already experienced changes (hormone levels as well as immunological) in their pregnancy in comparison with primiparous women. The previous experienced changes in hormonal and immunological environment can lead to immune tolerance/adaptation and reduce the risk for preeclampsia [19, 20].

However, several studies have reported an association between prior abortion and an increased risk of preeclampsia in Ethiopia [6], Iran [5], China [8], and Scotland [21]. Interestingly, while one prior miscarriage was not found to be associated with preeclampsia, two or more miscarriages were found to be associated with increased risks of preeclampsia [7]. On the other hand, Holmlund et al. found no association between induced abortion and preeclampsia in Finland [9]. Moreover, Clark et al. reported no association between miscarriage and gestational hypertension, even in women with more than two abortions [10]. In a case–control study involving 200 cases and 100 controls, no significant difference was noted in preeclampsia/eclampsia between women with one prior abortion and those with one prior live birth [11]. Interestingly, no reduction in the prevalence of preeclampsia was noted following prior induced or spontaneous abortions [12].

It is worth mentioning that our results should be cautiously compared with those of previous studies. First, while most of these studies assessed the association between induced abortion and preeclampsia, we assessed the association between spontaneous abortion and preeclampsia. Perhaps, spontaneous and induced abortions are two different entities with different pathophysiologies. Because of traditional and religious factors, induced abortion is not practiced in Sudan, with few exceptions for medical reasons. Second, the present study was designed to assess the association between spontaneous abortion and preeclampsia, regardless of the number of miscarriages; however, in several other studies, the association between prior abortion and preeclampsia depended on the number of prior abortions. Third, we need to consider the differences in social and genetic factors among different communities, which may affect the etiology and pathophysiology of preeclampsia in the communities.

In previous studies, prior miscarriages were found to be associated with an increased risk of poor maternal and perinatal outcomes, such as placental dysfunction disorders, stillbirth, small-for-gestational-age fetuses, antepartum hemorrhage, preterm birth, and low birth weight [7, 22]. The association between prior abortion and increased risk of preeclampsia (contrary/opposite to our results) could be explained by the hypothesis that placental dysfunction could result in early placentation failure, with poor implantation and placentation, which are common features of both abortion and preeclampsia [23, 24]. Moreover, prior abortion may lead to maternal exposure to fetal cells and may induce maternal immune tolerance, which may play an important role in the development of preeclampsia [25].

There are many limitations in this study. Many factors, such as communicable diseases (e.g., herpes simplex virus type 2 infection and toxoplasmosis), were not assessed in the present study. These diseases have been reported to be associated with preeclampsia [26, 27], and they may be associated with abortion itself. We objectively assessed the history of spontaneous abortion in women with preeclampsia. The information regarding abortion and its history may not be accurate, as it depends on recall bias.

## Conclusion

The present study revealed that previous spontaneous abortion reduced the risk of preeclampsia by 59.0%.

## Data Availability

The datasets generated and/or analysed during the current study are not publicly available (because the manuscript is still under the peer review process) but are available from the corresponding author on reasonable request.
